# Supplementary material for: Conditional chemoconnectomics (cCCTomics) as a strategy for efficient and conditional targeting of chemical transmission
Source: eLife. 2024 Apr 30;12:RP91927. doi: 10.7554/eLife.91927 (PMC11060718; doi:10.7554/eLife.91927)
Supplement: Figure 3—source data 3. [file elife-91927-fig3-data3.zip › Figure 3 source data3 part 2.zip!/Figure 3-Source data 3-part2/1053-1-CRISPResso_Report_mpeqg9da.zip!/CRISPResso_on_1053_2/2a.Nucleotide_percentage_quilt.pdf]

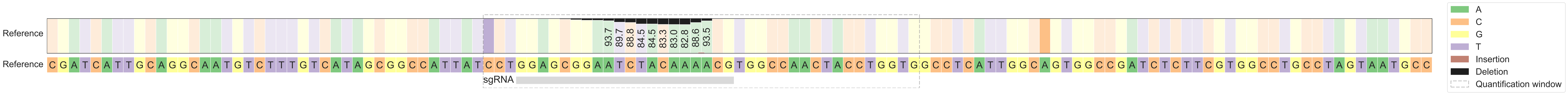

Reference
Reference
CGATCATTGCAGGCAATGTCTTTGTCATAGCGGCCATTATCCTGGAGCGGAATCTACAAAACGTGGCCAACTACCTGGTGGCCTCATTGGCAGTGGCCGATCTCTTCGTGGCCTGCCTAGTAATGCC
93.7
89.7
88.8
84.5
84.5
83.3
83.0
82.8
88.6
93.5
sgRNA
A
C
G
T
Insertion
Deletion
Quantification window
